# Rapid formation of carbon nanotubes–natural rubber films cured with glutaraldehyde for reducing percolation threshold concentration

**DOI:** 10.1186/s11671-024-03970-5

**Published:** 2024-02-19

**Authors:** Rawiporn Promsung, Arthittaya Chuaybamrung, Antonia Georgopoulou, Frank Clemens, Yeampon Nakaramontri, Jobish Johns, Nussana Lehman, Ladawan Songtipya, Ekwipoo Kalkornsurapranee

**Affiliations:** 1 Division of Physical Sciences, Faculty of Science, Prince of Songkla University, Hat-Yai, Thailand; 2 Department of Functional Materials, Empa˗Swiss Federal Laboratories for Materials Science and Technology, Dübendorf, Switzerland; 3 Sustainable Polymer and Innovative Composite Materials Research Group, Department of Chemistry, Faculty of Science, King Mongkut’s University of Technology Thonburi, Bangkok, Thailand; 4 Department of Physics, Rajarajeswari College of Engineering, Bangalore, India; 5 Center of Excellence in Bio-Based Materials and Packaging Innovation, Faculty of Agro-Industry, Prince of Songkla University, Hat-Yai, Thailand

**Keywords:** Natural rubber latex, Glutaraldehyde, Carbon nanotubes, Electrical conductivity, Composites

## Abstract

Carbon nanotubes (CNTs) filled natural rubber (NR) composites with various CNT contents at 0, 1, 2, 3, 4 and 5 phr were prepared by latex mixing method using glutaraldehyde as curing agent. This work aims to improve the electrical and mechanical properties of CNT filled NR vulcanizates. The CNT dispersion of NR composites was clarified using dispersion grader, optical microscopy and scanning electron microscopy. The electrical properties of NR composites in the existing of CNT networks were studied by following the well-known percolation theory. It was observed that the NR composites exhibited low percolation threshold at 0.98 phr of CNT. Moreover, a three-dimensional network formation of CNT in the NR composites was observed and it is indicated by the *t-*value of 1.67. The mechanical properties of NR composites in terms of modulus, tensile strength and hardness properties were increased upon the addition of CNT to the optimum mechanical properties at 1 phr of CNT. Therefore, the present work is found the novelty of the study that the conductive rubber latex film can be produced using GA as low-temperature curing agent which enhanced good electrical properties. Moreover, this work is found to be beneficial in case of conductive rubber latex film that requires high modulus at low strain. The additional advantage of this system is the curing process occurs at low-temperature using GA and it can be easily processed.

**Graphical abstract:**

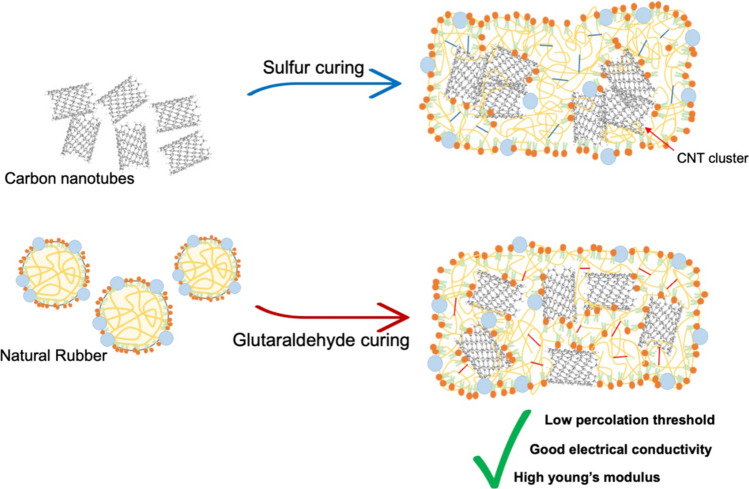

## Introduction

Natural rubber (NR) is an important elastomeric material due to its renewable resource and it is widely applied for several applications because of various outstanding properties such as strength, resilience, elongation at break and so forth [1]. However, unmodified NR also has some undesirable properties, particularly, low heat and abrasion resistances, poor ageing properties, sensitive to heat, low modulus and poor hardness [2]. Therefore, NR usages in case of some applications required a modification process in order to improve its specific properties and to reduce its drawbacks [3]. There are several ways to enhance the physical properties of NR, including, mixing of natural rubber with nanoscale fillers. The most commonly used fillers are silica, nanoclay, carbon nanotube (CNT), etc. The physical properties of NR can be effectively improved with the addition of nano-fillers due to their extraordinary properties at nano level. CNT has attracted great attention in composite technology applied in various industries due to their several unique properties including mechanical properties, excellent thermal conductivity, and outstanding electrical conductivity. This is due to CNT consists of a rolled graphene, the sp^2^-hybrid carbon layer, which forms cylinders with diameter in the order of nanometer and its length varies up to several millimeters [2].

The interactions between polymer–polymer, polymer–filler and filler–filler are essential to regulate the ultimate properties of nanocomposites. Dispersion of CNT is sturdily dependent on the interfacial interaction and mixing method [4]. Adding appropriate amounts of CNT with good dispersion can improve the properties of NR. The important properties of industrial interest are the improvement of strength, elasticity and electrical conductivity. However, to produce a homogeneous dispersion of CNT into NR, several factors must be considered specifically the mixing conditions and the molecular characteristic of the rubber matrix. This in turn promotes poor electrical conductivity and high percolation threshold concentration in the NR/CNT composites [5–7]. Nakaramontri et al*.* [8] prepared CNT filled NR composites using sulfur as curing agent by melt mixing and latex mixing methods. The CNTs were prepared without and with bis(triethoxysilylpropyl)tetrasulfide (TESPT). It was observed that the composites via latex mixing method showed low percolation threshold at 1.12 and 0.55 phr of CNT without and with TESPT, respectively. Kranoi et al. [9] developed NR/CNT composites using sulfur as a curing agent via latex mixing method. The CNT surfaces were functionalized with silver nanoparticle (AgNP) to compare the unfunctionalized CNT. It was observed that the percolation threshold of NR composites without and with AgNP are 3.64 and 2.92 phr, respectively. However, the preparation of CNTs based NR using sulfur as a curing agent still has limitations in vulcanization, which requires a lot of other chemicals, such as activators and accelerators. Nevertheless, irradiation vulcanization of rubber serves as a non-chemical alternative, although its popularity is limited by the relatively high production costs and environmental concerns associated with the irradiation source [10, 11].

Currently, a method involving low-temperature vulcanization of NR molecular chains using glutaraldehyde (GA) as a curing agent has been devised [12–14]. The primary advantage of this vulcanization system is its ability to vulcanize NR at low temperature (~ 50 °C) without the need for specific activators and accelerators. This method is environmentally advantageous and proves to be a cost-effective means of curing NR. This method is easy to process and uses less energy to prepare vulcanized rubber. The crosslinking process of NR with GA involves two consecutive steps. Initially, pentane-1,5-diylidenediamine is generated by the reaction of GA with ammonia present in the latex. Subsequently, the crosslinking occurs between NR molecular chains and pentane-1,5-diylidenediamine by ‘ene’ reaction. Additionally, several studies have reported that this curing system offers better thermal stability compared to the sulfur cured system [12–15].

Therefore, this work is aimed to study the preparation and properties of CNT filled NR composite by latex mixing method using GA curing system. To enhance the electrical and mechanical properties of NR composites involved the incorporation of CNT as a conductive filler at different CNT concentrations (0, 1, 2, 3, 4, and 5 phr). The electrical conductivity was studied to confirm the construction of CNT network in NR matrix and also compared with the other conductive materials. Mechanical properties and the dispersion of CNT filled NR composites were investigated by tensile testing, hardness, light microscopy and SEM, respectively.

## Experimental

### Materials

The high ammonia concentrated latex with 60% dry rubber content (DRC) was purchased from Chalong Latex Industry (Songkhla, Thailand). Glutaraldehyde (GA) was procured from Wing Great Industry Co., Ltd, (Bangkok, Thailand) [14, 15]. Carbon nanotube (CNT) with NC7000 grade was acquired from Nanocyl S.A. (Sambreville, Belgium) [8]. A multiwalled CNT with a diameter of 9.5 nm, a length of ~ 1.5 μm, and a purity of 90% has been used during the entire course of investigation. The sodium dodecyl sulfate (SDS) used to disperse CNT particles in the latex deionized water was manufactured by Banksia Scientific Company (Queensland, Australia) [8].

### Preparation of NR/CNT composites with different %DRC of NR latex by GA curing system

**Fig. 1:**
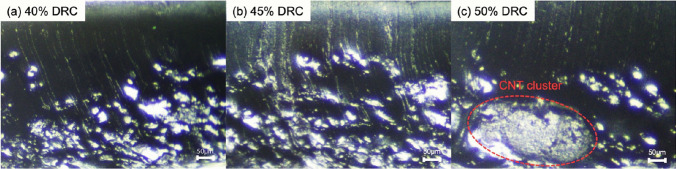
Optical microscope images (5.5×) of CNT (3 phr) filled NR vulcanizates using GA as curing agent with different %DRC of NR latex, **a** 40%DRC, **b** 45%DRC and **c** 50%DRC

NR latexes with 40, 45 and 50%DRC were prepared for the fabrication of nanocomposites. The CNT dispersion was prepared by adding 3 phr of CNT and 20wt% of SDS into deionized water to be used to dilute the %DRC of NR latex. Then, the CNT solution was sonicated using ultrasonicator probe for 10 min. The CNT dispersion and 12.5% GA aqueous solution were slowly added into NR latex during mechanical stirring at 200 rpm for 30 min. Then, the mixture was casted on glass plate and dried in a hot air oven at 50 °C for 24 h. The NR composite films were removed and introduced for morphological and mechanical characterizations. CNT filled NR vulcanizates were successfully prepared at different %DRC of NR latex using GA as a curing agent. Figure 1 shows the light microscope images of CNT filled NR vulcanizates at different %DRC of NR latex. It is clearly noticed that the NR vulcanizate at 50%DRC occurs cluster of CNT agglomeration as seen in Fig. 1c. This might be due to the lower deionized water content in CNT solution led to poor dispersion of CNT in NR latex. Table 1 summarizes the mechanical properties of CNT filled NR vulcanizates at different %DRC. It is found that the NR vulcanizate at 45%DRC exhibited the highest moduli and tensile strength. Therefore, according to the above morphological and mechanical properties, the NR vulcanizate from 45%DRC of NR latex has been selected for the preparation and to study the properties of CNT filled NR vulcanizates at different CNT contents.

**Table 1:** Mechanical properties of CNT (3 phr) filled NR vulcanizates using GA as curing agent with different %DRC of NR latex

DRC (%)	100% modulus (MPa)	300% modulus (MPa)	500% modulus (MPa)	Tensile strength (MPa)	Elongation at break (%)
40	1.61 ± 0.05	2.25 ± 0.08	4.68 ± 0.17	5.74 ± 0.25	577.18 ± 24.14
45	2.00 ± 0.03	2.83 ± 0.01	5.50 ± 0.19	6.20 ± 0.47	569.15 ± 18.85
50	1.18 ± 0.01	1.78 ± 0.03	4.37 ± 0.24	5.83 ± 0.20	571.86 ± 8.43

### Preparation of NR/CNT composites using GA as curing agent at various CNT contents

The CNT dispersion was prepared by adding CNT and 20wt% of SDS into deionized water to be used to dilute %DRC of NR latex. Then, the CNT solution was sonicated using ultrasonicator probe for 10 min. The CNT dispersion and a 12.5% GA aqueous solution were gradually introduced into NR latex while mechanically stirring at 200 rpm for 30 min. Subsequently, the mixture was cast onto a glass plate and dried in a hot air oven at 50 °C for 24 h [8, 14, 15]. The NR composite films were removed and introduced for morphological and mechanical characterizations. The CNT filled NR vulcanizates with various CNT contents such as 0, 1, 2, 3, 4 and 5 phr were prepared.

## Characterization

The electrical conductivity (σ) of the resulting NR/CNT composites was measured using an LCR meter (E4990A, Keysight Technologies, Inc., California, USA). A dielectric test fixture with two electrode plates (16451B dielectric test fixture, Keysight Technologies, Inc., California, USA) with 38 mm electrode diameter was connected to the LCR meter [8, 9]. The resistance (*Rp*) was measured in the range of frequency from 20 to 10,000 Hz. The electrical conductivity (σ) was estimated using the Eq. (1) [8, 9].

1 $$\sigma = \frac{1}{\rho } = \frac{d}{{\left( {R_{p} } \right) \cdot A}}$$

where *d* and *A* are the thickness of sample and the area of an electrode, respectively. The ρ value is the volume resistivity [8, 9].

The universal testing machine (model H10KS, Hounsfield, UK) were applied to measure the tensile properties of composites based on NR vulcanizates [14, 15]. The tests were carried out with a crosshead speed of 500 mm/min at room temperature using dumbbell shaped specimens according to ASTM D412 [14, 15]. For hardness properties, the samples were tested using a Shore A durometer (Frank GmbH, Hamburg, Germany) as per ASTM D2240 [14, 15]. The mechanical properties, including modulus, tensile strength, elongation at break, and hardness, were determined based on the average of five test results.

To investigate filler–filler interaction, the composites were elucidated by hysteresis and swelling experiments. The hysteresis behavior was performed by the dynamic tensile testing using 10 cycles between 0 and 150% strain with a speed of 200 mm/min at room temperature [16]. The crosslink density of the NR composites were determined by the swelling method. The composites were elucidated by soaking the specimen pieces of 10 × 10 × 2 mm^3^ in toluene in a closed system for 72 h at room temperature [17]. The samples were weighed before and after soaking. The crosslink density was calculated using the Flory–Rehner equation [17] (Eq. 2) as shown below:

2 $$\rho_{c} = - \frac{1}{{2V_{s} }}\frac{{In\left( {1 - V_{r} } \right) + V_{r} + \chi_{1} (V_{r} )^{2} }}{{(V_{r} )^{1/3} - V_{r} /2}}$$

where $\rho_{c}$ is crosslink density, *V*_s_ is the molar volume of toluene (106.9 cm^3^ mol^−1^ at 25 °C), $\chi_{1}$ is the interaction parameter and *V*_r_ can be determined from Eq. (3).

3 $$\frac{{V_{r}^{o} }}{{V_{r} }} = 1 - \left[ {3C\left( {1 - V_{r}^{{o^{1/3} }} } \right) + V_{r}^{o} - 1} \right]\frac{\emptyset }{1 - \emptyset }$$

where $V_{r}^{o}$ is the rubber fraction in the swollen gel, *C* is the parameter for the rubber interaction (*C* = 1.17) and ϕ is the volume fraction of rubber.

Morphological properties of NR vulcanizates were investigated by an optical microscopy (Carl Zeiss Microscopy GmbH, Oberkochen, Germany). The samples were fast cut with a razor blade (Energizer^®^ Holdings, Inc., Missouri, USA) to create smooth sample surfaces for optical microscopy before the investigation. To confirm the agglomeration of CNT filled NR, the surfaces of composite samples were scanned by SEM (VEGA3, Tescan, Czech Republic) with an accelerating voltage of 15 kV. The fractured surfaces of NR vulcanizates were sputter coated with gold before introducing in to morphological studies. To examine the dispersion of CNT in NR matrix, disperse grader testing were performed according to ASTM D 7723 by using Alphaview Dispergrader (Alpha Technologies, USA) incorporated with ShuttleXpress software to analyze %dispersion of the composite. According to the standard [18], illumination was at 30º to the sample surface.

## Results and discussion

### Electrical conductivity

The electrical conductivity of CNT filled NR vulcanizates with various CNT contents can be associated to the formation and the dispersion of CNT network in NR composites, which is demonstrated by the percolation threshold (*f*_*c*_) which is the critical CNT content to form filler network. The percolation threshold can be estimated according to the fundamental percolation theory [8, 9] using Eq. (4) and (5)

4 $$\sigma_{DC} = k\left( {f - f_{c} } \right)^{t} , f > f_{c}$$

5 $$log(\sigma_{DC} ) = logk + t \cdot {\text{log}}\left( {f - f_{c} } \right)$$

where *σ*_*DC*_ is the electrical conductivity of composite and *k* is a constant. The parameters *f* and *f*_*c*_ are the volume fraction of CNT and the volume fraction at the percolation threshold, respectively. The *t-*value is the fitting parameter which indicates the three-dimensional networks (3D network) of CNT in rubber matrix lies in between 1.6 and 2.0, while the value of *t* lies below 1.6 indicates two-dimensional (2D network) conductive networks [8, 9].

**Fig. 2:**
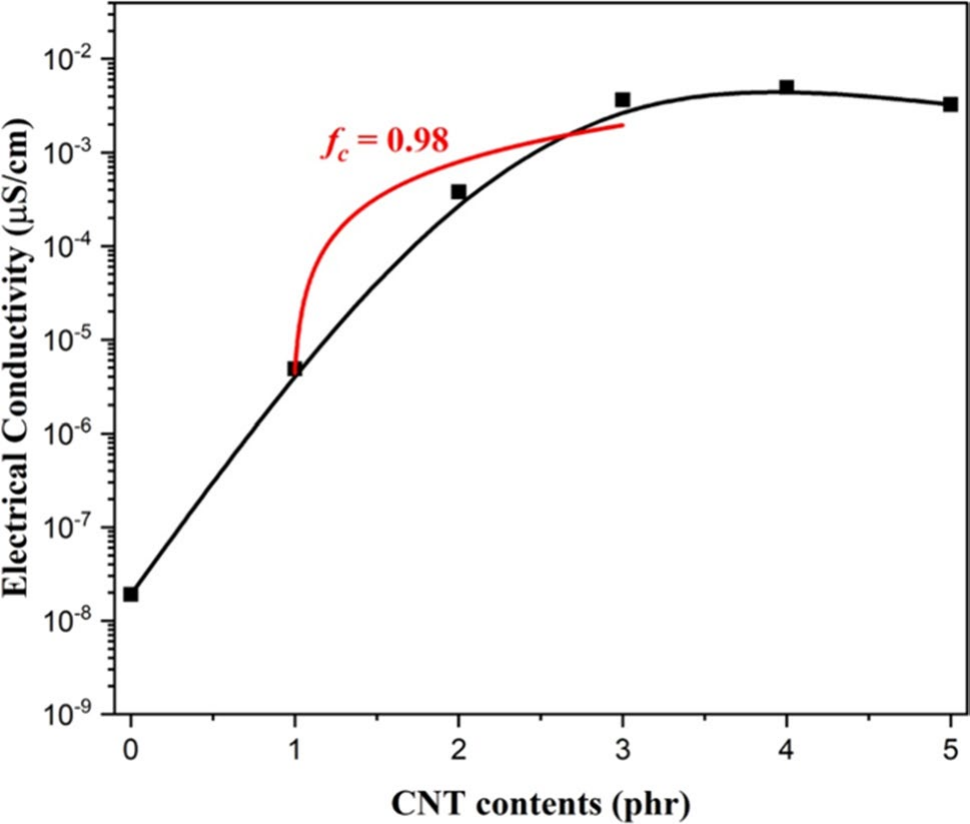
Electrical conductivity of NR/CNT composites using GA as curing agent

**Fig. 3:**
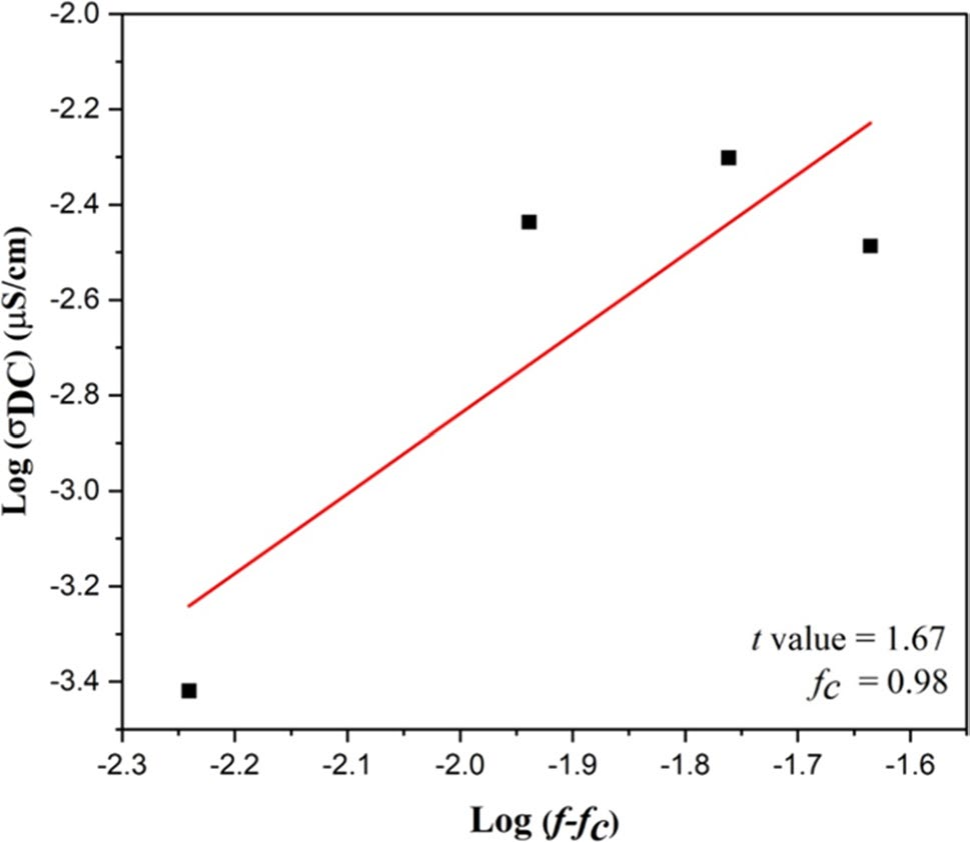
Plot of log (σ_DC_) versus log (*f*–*f*_*c*_) of the CNT filled NR vulcanizates

**Fig. 4:**
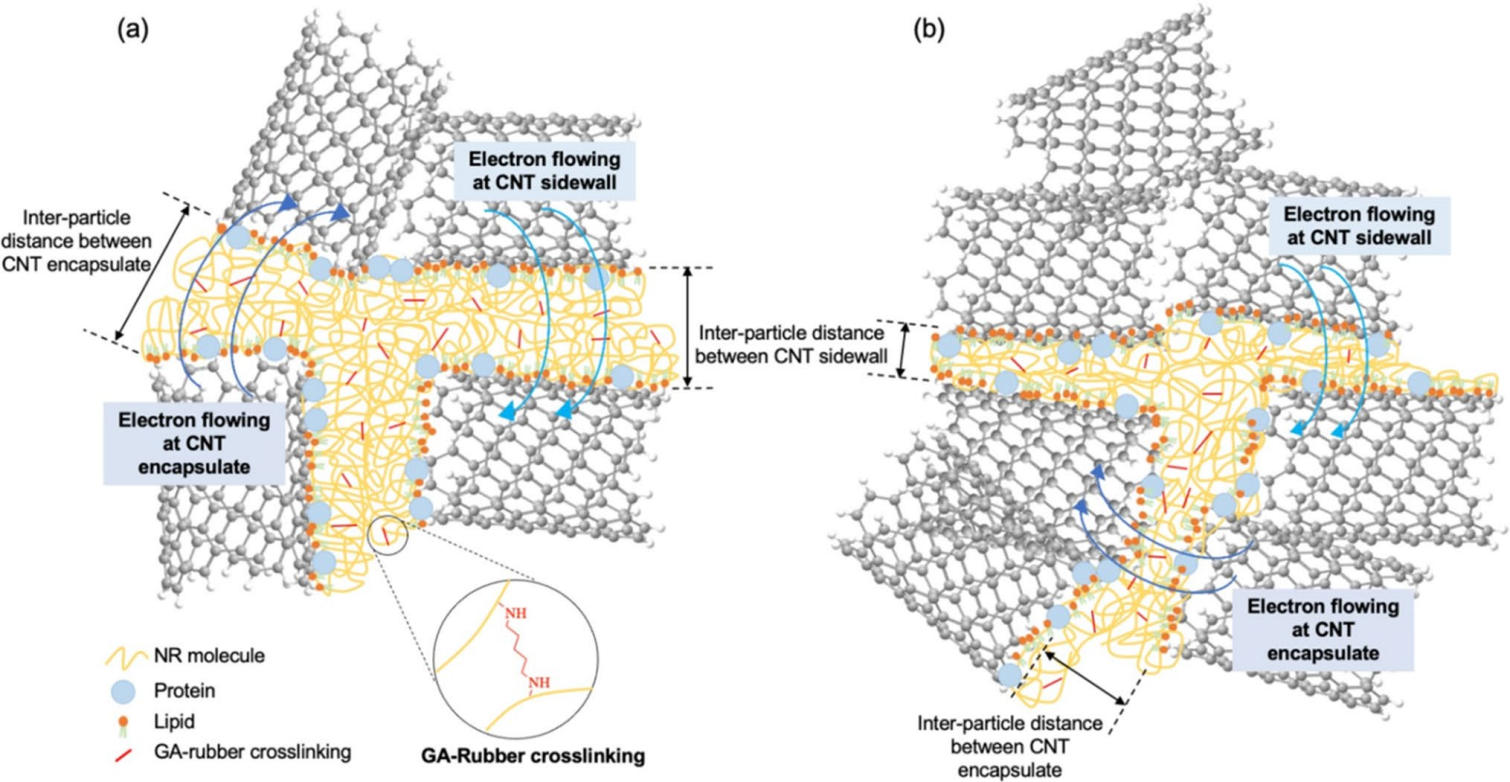
A proposed model of electron flowing through the CNT pathway at **a** 1 phr and **b** above 1 phr of CNT filled NR composites using GA as curing agent

Figures 2 and 3 showed the electrical conductivity and the plot of log(*σ*_*DC*_) versus log(*f-f*_*c*_) of CNT filled NR composites, respectively. It is observed that the electrical conductivity of CNT filled NR composites increase at 1 phr of CNT loading (*σ*_*DC*_ = 10^–5^ µS/cm) up to 3 phr and reaches a constant electrical conductivity of 10^–3^ µS/cm. The increasing of electrical conductivity can be attributed to the distribution of CNT in the NR matrix [19]. This is also due to the lower CNT content (< 1 phr), where the CNT pathway to conduct electron current might be hindered by the rubber matrix as a result of larger inter-particle distance between CNTs [19–21]. On the other hand, the NR composites at higher CNT contents (> 1 phr) have shorter distance between CNT pathway led to increase the electrical conductivity due to the ease of electron flow through the CNT pathway [20, 22] as explained in Fig. 4. Furthermore, the constant *σ*_*DC*_ values are summarized in Table 2. The *σ*_*DC*_ of several rubber composites are observed in the range of 10^–4^–10^2^ µS/cm. Therefore, the CNT filled NR composites using GA curing agent showed the conductivity in the same range of others rubber composites. According to the Eqs. (2) and (3), the percolation threshold and the *t-*value of CNT filled NR vulcanizate are 0.98 phr and 1.67, respectively. Therefore, the CNT filled NR composites cured with GA prepared by film casting method exhibited the 3D network formation of CNT in rubber matrix at lower percolation threshold when compared to the other rubber-based composites with different fillers using sulfur curing system as summarized in Table 2.

**Table 2:** The percolation threshold and *t-*value of rubber composites

Materials	Curing agent	Percolation threshold (phr)	*t*-value	Constant EC	Ref.
NR latex/CNT	GA	0.98	1.67	10^−3^	This work
NR/CNT	S	3.10	0.60	10^−2^	[8]
NR/m-CNT^*a^		1.14	1.70	10^−3^	
NR/CNT-L^*b^		1.12	1.40	10^−3^	
NR/m-CNT-L		0.55	1.79	10^−3^	
NR/CNT-L	S	3.64	2.34	10^−4^	[9]
NR/CNT/AgNP^*c^-L		2.92	1.86	10^−1^	
NR/CNT	S	3.00	1.40	10^0^	[21]
NR/CNT/CCB^*d^		2.00	1.70	10^0^	
NR/m-CNT/CCB		1.00	1.80	10^1^	
ENR/CNT		1.50	1.60	10^0^	
ENR/CNT/CCB		0.50	1.80	10^1^	
ENR/m-CNT/CCB		0.30	2.00	10^2^	
ENR/CNT	S	1.20	2.30	10^−2^	[23]
ENR/CCB		12.00	2.50	10^−4^	
IR/CNT	S	1.00	2.20	10^−1^	[20]
IR/CNT/CCB		0.70	2.25	10^0^	
ENR/CNT		1.85	2.20	10^0^	
ENR/CNT/CCB		0.50	1.95	10^1^	
NR/CNT		2.50	1.50	10^0^	
NR/CNT/CCB		2.00	1.70	10^1^	
DPNR/CNT		2.50	1.50	10^−1^	
DPNR/CNT/CCB		2.00	1.70	10^1^	
ENR/CNT	S	2.30	1.74	10^1^	[24]
NR/CNT	S	3.64	2.34	10^−4^	[25]
NR/CNT/IL^*e^		2.92	2.32	10^−1^	
NR/CNT	S	2.00	1.62	10^−1^	[25]
NR/CNT/CCB		1.00	2.13	10^2^	
NR/CNT	S	3.10	0.60	10^−2^	[19]
ENR/CNT		0.40	1.50	10^−2^	
MNR/CNT		0.60	1.00	10^−2^	

*ENR* is epoxidized natural rubber, *IR* is isoprene rubber, *DPNR* is deproteinized natural rubber, *MNR* is maleated natural rubber

^*a^m-CNT is the modified CNT or functionalized CNT

^*b^CNT-L is the filler dispersion by using latex mixing method

^*c^AgNP is silver nanoparticles

^*d^CCB is conductive carbon black

^*e^IL is ionic liquid

### Mechanical properties

**Fig. 5:**
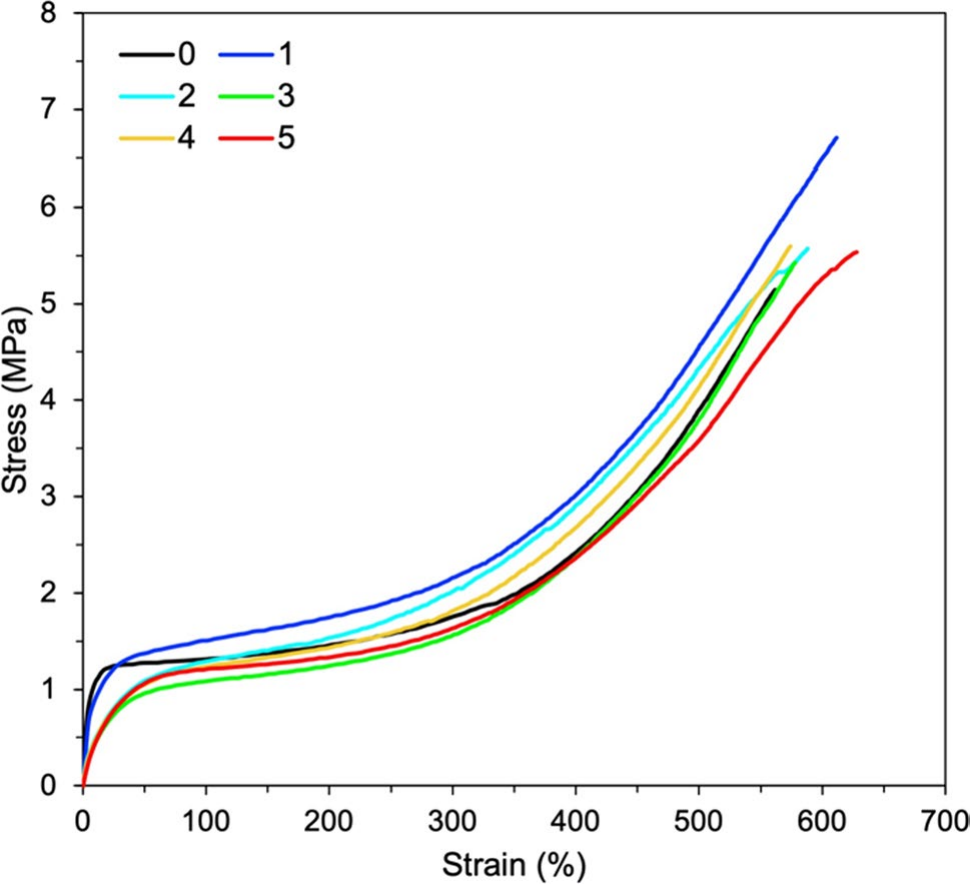
Stress–strain curves of NR/CNT composites using GA as curing agent

The tensile properties of CNT filled NR composites are investigated in terms of modulus, tensile strength and elongation at break. Figure 5 shows the stress–strain curves of NR/CNT composites cured with GA. There is a significant change in the deformation behavior of cured NR upon the addition of CNT. At the initial stage of strain (lower than 100%), the composites showed drastic increase in stress following the Neo-Hookean theory. This might be due to the chain entanglement of NR molecules, which can refer to the strength of the composites against extension [26]. Above 300% strain, the stress of NR/CNT composites increased again until reaching a maximum stress. This behavior indicates the strain-induced crystallization of the NR/CNT composites [15, 26]. Table 3 summarizes overall mechanical properties of NR/CNT composites using GA as curing agent. It is observed that addition of CNT showed the moduli, tensile strength and elongation at break higher than the NR film without CNT. This is due to the incorporation of CNT with uniqueness properties that enhances the strength and the stiffness along with the flexibility of NR/CNT composites [2]. However, tensile strength and elongation at break of NR/CNT composites are found to be decreased above 1 phr of CNT due to the agglomeration of CNT in NR matrix [27]. The hardness values of NR/CNT composites using GA as curing agent are also summarized in Table 3. It is clearly seen that the hardness value of the composites is increased upon increasing the CNT content. This is due to the incorporation of CNT nanofillers into the NR matrix by reducing the elasticity of NR chains and makes the composite more rigid. It results in an increased hardness on increasing the CNT contents [2].

**Table 3:** Mechanical properties of NR vulcanizates with different CNT contents using GA as curing agent

CNT contents (phr)	100% modulus (MPa)	300% modulus (MPa)	500% modulus (MPa)	Tensile strength (MPa)	Elongation at break (%)	Hardness (Shore A)
0	1.29 ± 0.03	1.77 ± 0.02	3.98 ± 0.11	5.07 ± 0.10	555.76 ± 8.14	47.80 ± 2.39
1	1.35 ± 0.07	1.97 ± 0.17	4.43 ± 0.20	6.24 ± 0.41	588.41 ± 21.76	48.00 ± 0.71
2	1.33 ± 0.08	1.97 ± 0.14	4.20 ± 0.17	5.64 ± 0.65	585.96 ± 35.69	50.80 ± 0.45
3	1.31 ± 0.14	2.16 ± 0.15	3.85 ± 0.07	5.18 ± 0.39	509.55 ± 60.89	54.20 ± 1.30
4	1.33 ± 0.08	2.07 ± 0.14	4.07 ± 0.11	5.33 ± 0.24	553.32 ± 65.24	54.80 ± 0.84
5	1.18 ± 0.08	1.68 ± 0.06	3.85 ± 0.26	5.43 ± 0.41	601.54 ± 23.27	54.00 ± 1.00

**Fig. 6:**
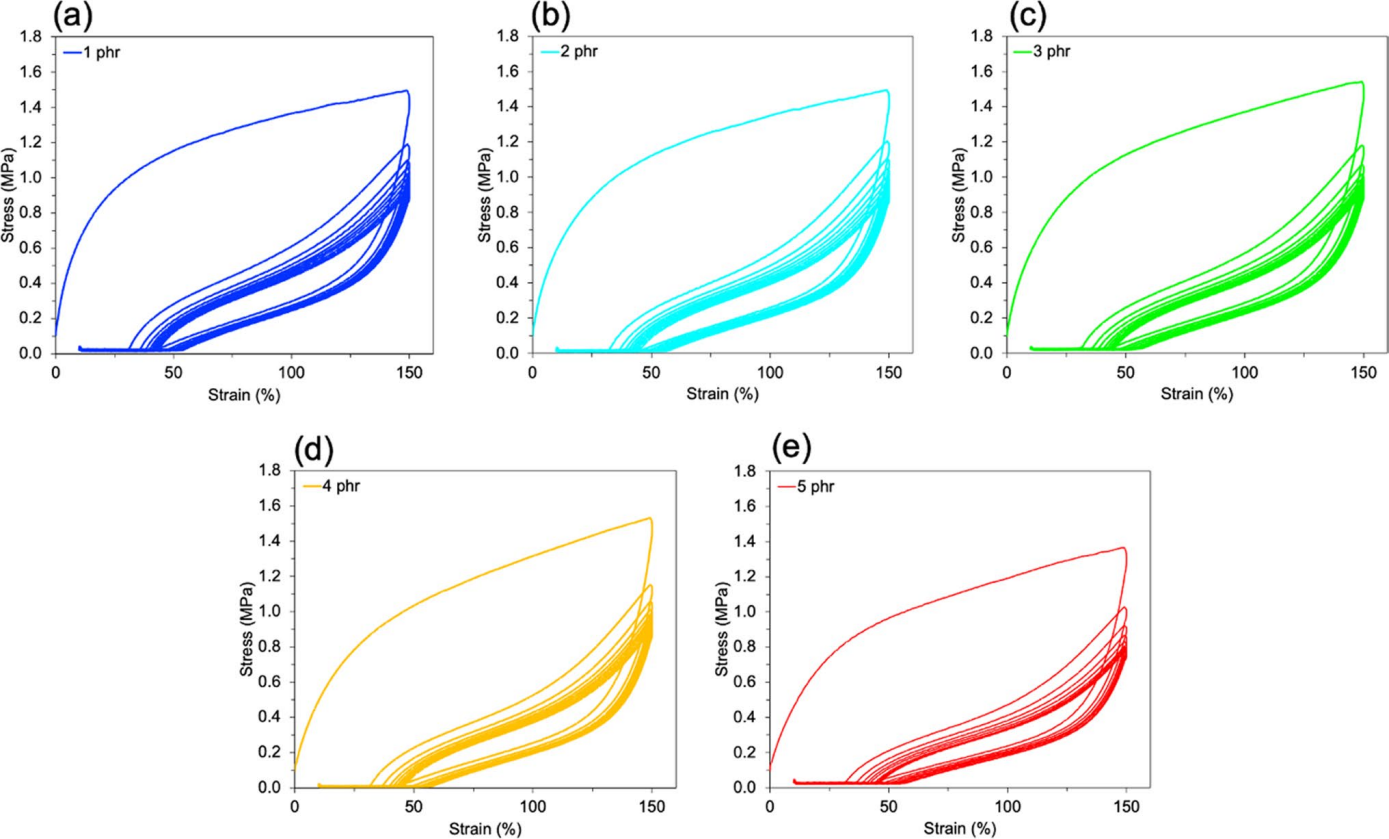
Stress–strain curves of NR/CNT composites during the 10 cycles of the dynamic tensile testing between 0 and 150 strain%. **a** 1 phr, **b** 2 phr, **c** 3 phr, **d** 4 phr and **e** 5 phr

Furthermore, the filler–filler interaction of the NR/CNT composites were elucidated by the dynamic tensile testing. Figure 6 displays stress–strain curves of NR/CNT composites during the 10 cycles of the dynamic tensile testing between 0 and 150% strain. It is observed that the maximum stress (σ_max_) of the NR/CNT composites significantly decreased after the 1st cycle of dynamic tensile test. This is due to the dissociation of filler–filler interaction of CNT led to the decreasing of σ_max_ in each cycle [16]. Additionally, to investigate the hysteresis behavior of the NR/CNT composites, the difference of σ_max_ of the 1st–2nd cycles and 1st–10th cycles were evaluated as summarized in Table 4. It was found that the difference σ_max_ of the composites with CNT above 1 phr observed higher Δσ_max_ than that composite with 1 phr of CNT. This attributed to the higher energy dissipation caused by the filler–filler interaction in the NR/CNT composites [16]. This hysteresis behavior is efficiency relating well the electrical conductivity results that strongly increased of the composites above 1 phr. On the contrary, the higher filler–filler interaction resulting in decreased of mechanical properties due to the self-agglomeration of CNT [28]. Moreover, crosslink density of the NR/CNT composites were evaluated using swelling method, the results were summarized in Table 4. It was found that the crosslink density continuously increased with increased CNT contents due to the additional rubber–filler interactions of the NR/CNT composites [29]. The higher crosslink density led to decreasing of tensile stress of the NR/CNT composites due the inter-crosslink chains restrict the orientation of the stretched inter-crosslink chains [16], consequently, larger difference σ_max_ of the NR/CNT composites from hysteresis testing as well as decrease tensile strength from tensile testing.

**Table 4:** The hysteresis and swelling experiments of NR/CNT composites

CNT contents (phr)	Hysteresis experiment		Swelling experiment
	Δσ_max_ (MPa), 1st–2nd cycles	Δσ_max_ (MPa), 1st–10th cycles	Crosslink density (× 10^–5^) (mol/cm^3^)
1	0.24 ± 0.03	0.47 ± 0.04	2.29 ± 0.29
2	0.30 ± 0.04	0.59 ± 0.05	2.77 ± 0.54
3	0.33 ± 0.01	0.59 ± 0.03	3.01 ± 0.45
4	0.35 ± 0.01	0.63 ± 0.04	3.23 ± 0.37
5	0.32 ± 0.05	0.57 ± 0.02	2.74 ± 0.15

### Morphological properties

**Fig. 7:**
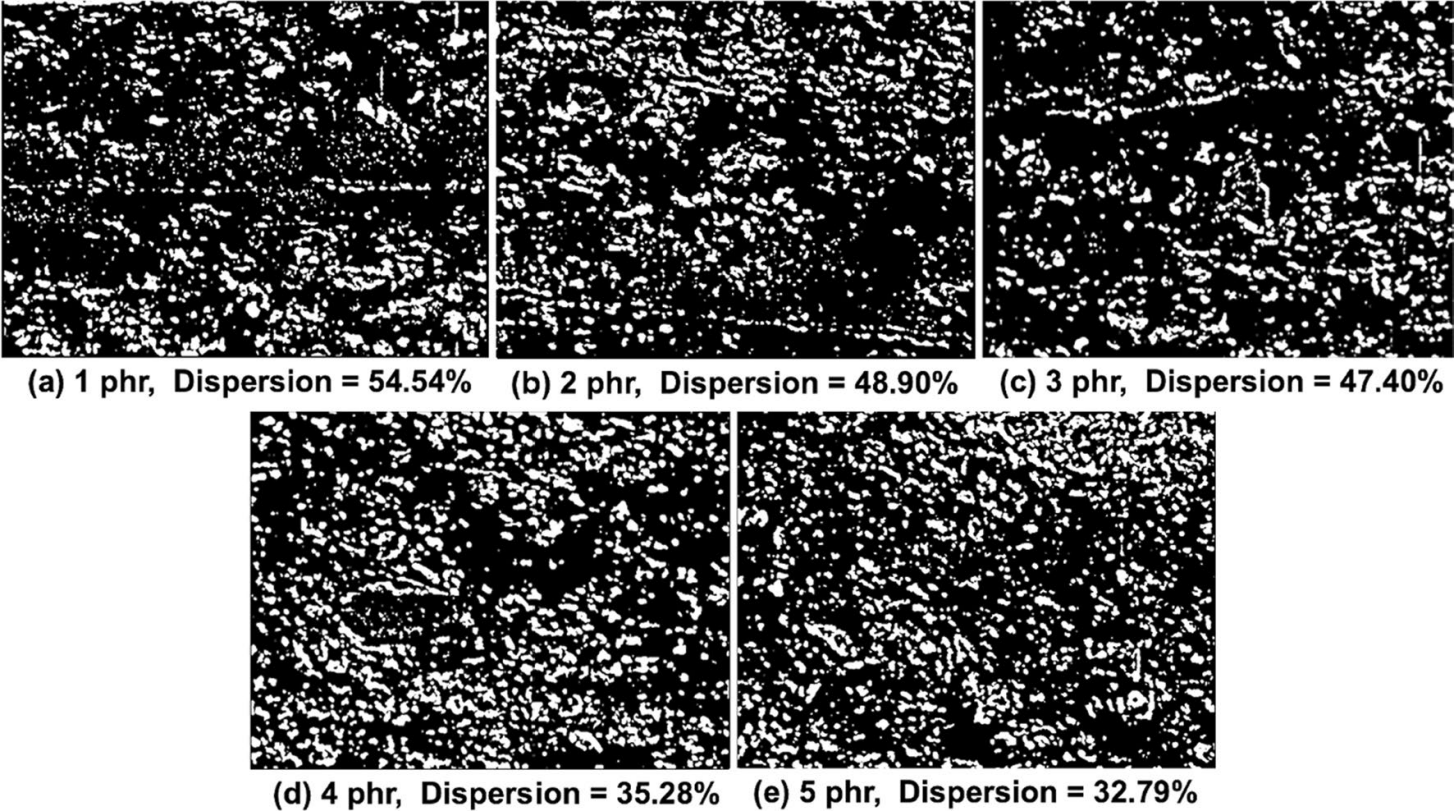
Dispersion Grader diagrams of NR/CNT composites. **a** 1 phr, **b** 2 phr, **c** 3 phr, **d** 4 phr and **e** 5 phr

In order to clarify the changing role of NR composite upon increasing the CNT contents, disperse grader testing were performed in term of diagrams and %dispersion as shown in Fig. 7. The bright spots in the images, corresponding to filler inclusions. Conversely, the regions of rubber matrix are depicted in dark color [18]. Large bright area indicates insufficiently dispersed fillers which are referred to as agglomerates. In addition, the %dispersion was analyzed from the bright and dark area by incorporated with ShuttleXpress software. It was found that the %dispersion of the NR/CNT composites continuously decreased with increased CNT loading, specially, adding CNT above 1 phr observed the dispersion lower than 50%. This attributed to the agglomeration of CNT caused by the higher filler–filler interaction of the composites.

**Fig. 8:**
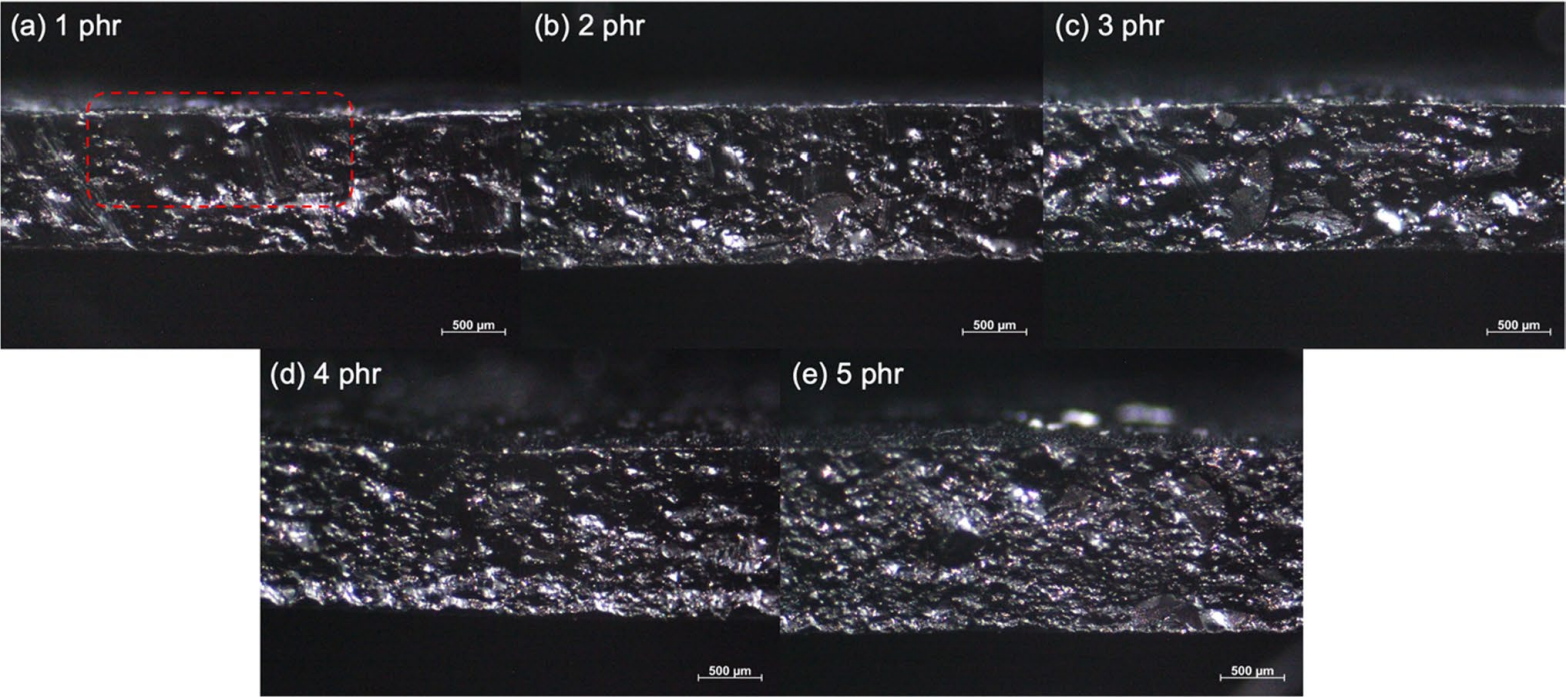
Optical microscope images (3.2×) of CNT filled NR vulcanizates at various CNT contents, **a** 1 phr, **b** 2 phr, **c** 3 phr, **d** 4 phr and **e** 5 phr

**Fig. 9:**
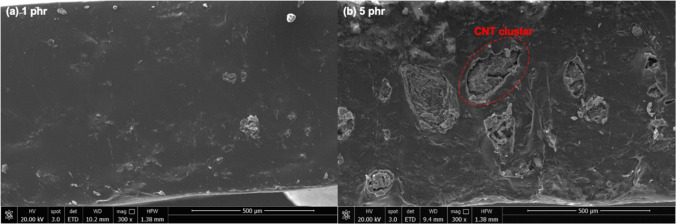
SEM images of CNT filled NR vulcanizates at various loadings of **a** 1 phr and **b** 5 phr at resolution magnification of 300×

**Fig. 10:**
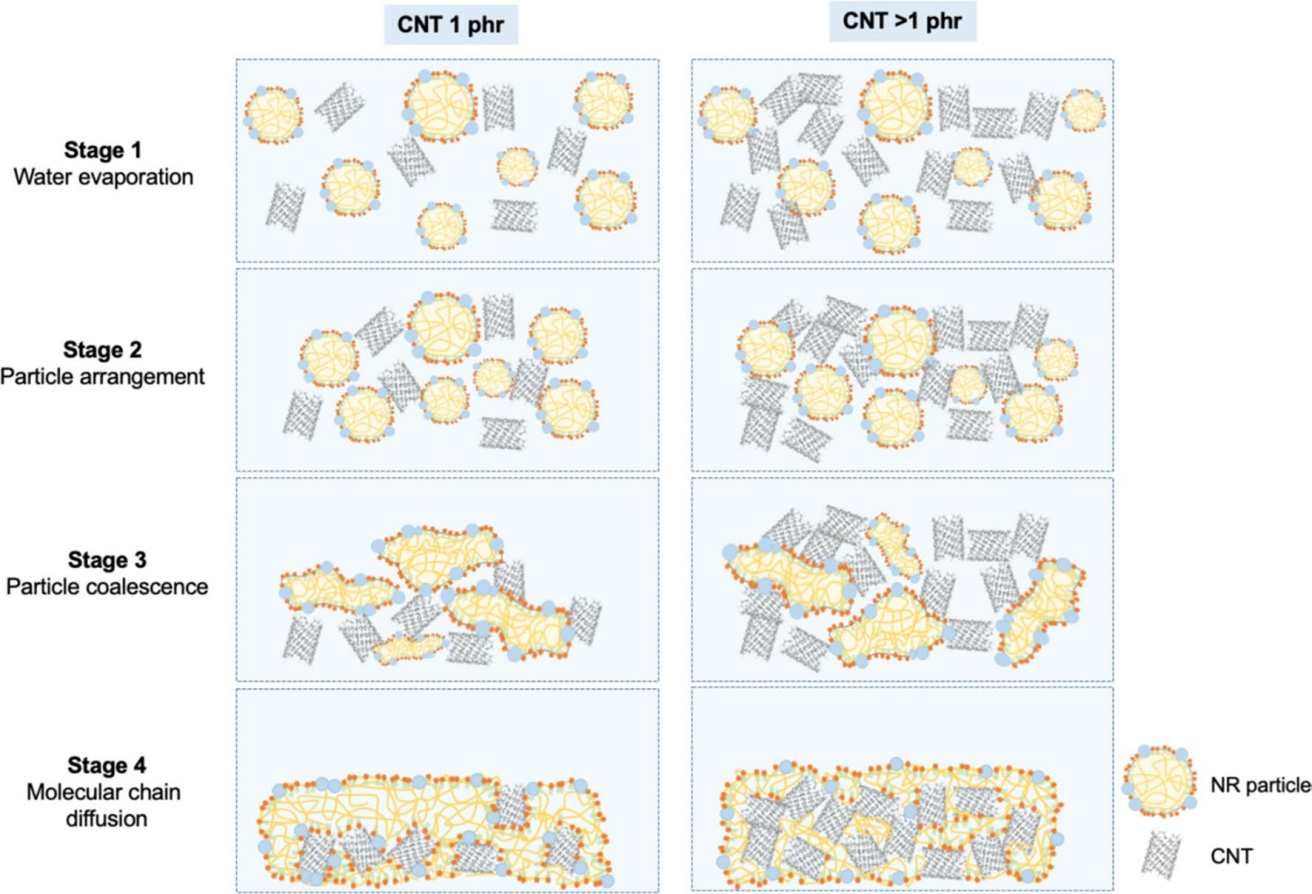
A proposed model of the film formation mechanism of CNT filled NR latex using GA as curing agent

The morphology of the composites are studied in terms of OM and SEM images as displayed in Figs. 8 and 9, respectively. Figure 8 showed the optical microscopy images of CNT filled NR vulcanizates at various CNT contents. In case of NR vulcanizate with 1 phr of CNT, the separation of CNT and NR layers can be clearly seen as noticed in Fig. 8a. This might be due to the delay in latex film formation of the NR latex [30]. After the evaporation of water from the latex mixture during film formation, as proposed in Fig. 10, CNT particles re-arrange and separate the NR matrix during the particle coalescence and the molecular chain diffusion stages especially at lower CNT content that results in the separation of CNT in NR matrix. Moreover, the addition of SDS in the latex curing system exhibited more stability in the NR latex by increasing the delay time for vulcanization in this system [31]. However, at the higher CNT content (above 1 phr), the NR vulcanizates showed slightly separate layer of CNT and NR matrix until 5 phr of CNT as shown in Fig. 8b–e. This is due to the higher CNT content in NR matrix which inhibited the separation of CNT and NR layers. In addition, the measurement of smaller areas with high resolution based on the SEM micrographs is presented as seen in Fig. 9. It is well correlated with the optical microscopy images as the dispersion of CNT is observed at 1 phr (Fig. 9a) and 5 phr (Fig. 9b). Higher level of agglomeration of CNT cluster is observed in the morphologies above 5 phr of CNT as shown in Fig. 9b. Furthermore, at higher CNT level, the agglomeration of CNT occurs that led to the decrease in the mechanical properties and increase in the electrical conductivity as mentioned above.

## Conclusion

The composites of CNT filled NR vulcanizates with various CNT contents were prepared by latex mixing method using GA as curing agent. The properties in terms of electrical conductivity, moduli, tensile strength, hardness, hysteresis behavior and swelling were found to be increased upon the addition of CNT. Moreover, the NR composites showed the low percolation threshold at 0.98 phr of CNT with a *t-*value of 1.67 that indicated the three dimensional network formation of CNT following the percolation theory. The dispersion of CNT was confirmed by dispersion grader, optical microscopy and SEM images. Therefore, it can be summarized that the CNT filled NR vulcanizates using GA as a low-temperature curing agent is a new method to prepare conducting rubber film. This method of preparation consumes less energy and the ease of processing without any specific activator and accelerator, which is highly favorable to the environment. Moreover, this system can be definitely introduced in multiple industries for the preparation of conducting rubber in cases of medical devices, military applications and soft robot sensor applications.

## Data Availability

Data sets generated during the current study are available from the corresponding author on reasonable request.
